# Exploring Undergraduate Pharmacy Students Perspectives Towards Antibiotics Use, Antibiotic Resistance, and Antibiotic Stewardship Programs Along With the Pharmacy Teachers’ Perspectives: A Mixed-Methods Study From Pakistan

**DOI:** 10.3389/fphar.2021.754000

**Published:** 2021-11-08

**Authors:** Faiz Ullah Khan, Amjad Khan, Shahid Shah, Khezar Hayat, Abubakar Usman, Farman Ullah Khan, Zakir Khan, Yusuf Karataş, Tawseef Ahmad, Jie Chang, Usman Rashid Malik, Asad Khan, Sundus Shukar, Muhtar Kadirhaz, Yu Fang

**Affiliations:** ^1^ Department of Pharmacy Administration and Clinical Pharmacy, School of Pharmacy, Xi’an Jiaotong University, Xi’an, China; ^2^ Center for Drug Safety and Policy Research, Xi’an Jiaotong University, Xi’an, China; ^3^ Shaanxi Center for Health Reform and Development Research, Xi’an, China; ^4^ Research Institute for Drug Safety and Monitoring, Institute of Pharmaceutical Science and Technology, Western China Science and Technology Innovation Harbor, Xi’an, China; ^5^ Department of Pharmacy, Quaid-i-Azam University, Islamabad, Pakistan; ^6^ Department of Pharmacy Practice, Faculty of Pharmaceutical Sciences, Government College University, Faisalabad, Pakistan; ^7^ Institute of Pharmaceutical Sciences, University of Veterinary and Animal Sciences, Lahore, Pakistan; ^8^ Discipline of Clinical Pharmacy, School of Pharmaceutical Sciences, Universiti Sains Malaysia, George Town, Malaysia; ^9^ Department of Pharmacology, Institute of Health Sciences, Çukurova University, Adana, Turkey; ^10^ Department of Pharmacy, COMSATS University Islamabad, Abbottabad, Pakistan

**Keywords:** antibiotics use, antibiotics resistance, Pakistan, pharmacy students, pharmacy teachers

## Abstract

**Background:** Antibiotic resistance (ABR) is one of the major issues around the globe. Timely education and awareness of pharmacy students regarding the appropriate use of antibiotics, ABR, and antimicrobial stewardships are required.

**Methods:** The present study was first conducted in 12 (public and private sector) universities among undergraduate pharmacy students (UGPS) (*n* = 414) irrespective of their study year through a validated questionnaire, and the insights of pharmacy teachers were taken through in-depth semi-structured interviews in the second phase. For the quantitative data, different statistical methods were used, and data were presented in tabulated form, whereas inductive thematic interpretation was used to categorize themes and derive conclusions from qualitative evidence.

**Results:** The majority of the students were males (*n* = 223, 54%) with the mean age group 19–23 years, and 20 faculty members were interviewed with a mean duration of 15 min. Students have good knowledge about antibiotics use and the majority purchased antibiotics through prescription (*n* = 277, 66.9%) during the last month and strongly agreed to stop unnecessary household storage (*n* = 183 44.2%). Most of the students have heard the terminologies related to antimicrobial resistance through social media while unaware (*n* = 104, 25.1%) of a Pakistan national action plan against AMR (antimicrobial resistance). Overall, respondents have a somewhat good understanding of the ABR. Regular use of antibiotics without consultation of a physician can lead to ABR and some wrong answers were observed (162, 39.1%; *p* > 0.05). The majority of the students (*n* = 198, 47.8%) and teachers believe that the current pharmacy syllabus must be swiftly updated with the new subjects related to ABR and AMS (antimicrobial stewardship) in Pakistan. The UGPS have emphasized (*n* = 220, 53.1%; Median = 1, IQR = 2) establishing a link between academia and hospitals. The ABR issue has been highlighted by pharmacy faculty members, who have urged students to take practical efforts toward ABR and AMS knowledge.

**Conclusion:** The UGPS knowledge related to ABR and AMS must be updated. Students at the undergraduate level must get training in order to encourage the sensible use of antibiotics. Courses on ABR and AMS should be included in present pharmacy curricula.

## Introduction

Antibiotics are used to treat a variety of infections because of their inherited capacity to prevent pathogenic microorganisms from growing or killing them (Martin et al., 2020). ABR has emerged as a result of the continued irrational and injudicious use of antibiotics. ABR is a silent wave that is sweeping the globe (Carvalho, 2021). Because of the subsequent rise in healthcare costs, it has important consequences (Founou et al., 2017; Carvalho, 2021). In the United States (US), nearly 2 million people are infected by resistant infections per year, resulting in the deaths of 23,000 patients and a loss of 55 billion dollars (Hayat et al., 2021). Similarly, annually in Europe, 25,000 people die, and there is a loss of 1.5 billion euros (Sarfraz et al., 2020; García et al., 2021). Owing to the lack of modern antibiotics, the devastating results attributed to ABR are continually rising (Hayat et al., 2021). Management of Resistant infections consumes a huge capital of 1.68 billion USD per year (Hayat et al., 2019a) which may surge to 3.5 billion USD per year in the next decade (OECD, 2019; Hofer, 2019). Approximately 47% of the antibiotics in Australia and almost half of the antibiotics in the US proved to be unjustified and used irrationally by the patients (Ingram et al., 2012; CDC., 2017). A WHO (world health organization) report revealed that out of a total of 67% of antibiotics used in the community about 30% are used irrationally by healthcare providers (Koivusalo and Mackintosh, 2008). There is a direct link between the use of antibiotics and ABR and their use in the community (Khan et al., 2020a; Khan et al., 2021; Malik et al., 2021).

Overall, different factors are involved in the spread of ABR, some of which are unique to both developing and developed countries. In lower middle income countries (LMICs) the most prevalent factors are; inadequate monitoring of resistance pattern, antibiotics quality standard, and availability without a prescription. LMICs are highly affected by ABR (Chokshi et al., 2019). In Pakistan, the use of antibiotics is regulated by registered prescribers and pharmacists where they used to prescribe antibiotics for bacterial infections (Hayat et al., 2021). In Pakistan, for a registered pharmacist, qualifying from the 5 years degree Viz. Doctor of Pharmacy (PharmD) is compulsory (Hayat et al., 2021). Antibiotic use among pharmacy students and teachers is still understudied.

Pharmacists have always been the frontline healthcare providers in the responsible use of antibiotics. Pharmacists being the custodians of the medication management system are having more knowledge than other healthcare providers and can enhance the rational use of antibiotics and control of infections (Dellit et al., 2007). Proper education and training of pharmacy students and teachers can change the prescribing patterns of antibiotics thus decreasing AMR (Burns et al., 2020). This study aimed to explore the role of pharmacy students and teachers in antibiotic resistance and antibiotic stewardship programs in Pakistan. A sharp decrease in AMR and an increase in positive outcomes of antibiotics use were observed by the involvement of pharmacists in antimicrobial therapy projects (Fay et al., 2019). However, in countries like Pakistan, China, and India, the role of a pharmacist is reported unsatisfactory in assuring the rational use of antibiotics (Shi et al., 2020). The unsatisfactory role of a pharmacist in these countries is mainly due to the lack of professional knowledge and awareness (Shi et al., 2020). Proper awareness campaigns, workshops, and educational training among pharmacists can help to improve their roles in providing rational antimicrobial therapies by decreasing AMR and increasing treatment outcomes (Ashraf et al., 2020). Pakistan is also one of the developing countries facing ABR and knowledge gaps in all health care settings regarding ABR and AMS. Therefore, the present mixed-methods study was designed to investigate the undergraduate students’ level of knowledge related to ABR and AMS along with the in-depth interviews with the faculty members, particularly regarding their role in ABR and AMS plans in Pakistan.

## Methods

### Study Execution

Pakistan has a total population of 208 million as per the 2017 census with a 2.4% population growth rate (Goujon et al., 2020). In Pakistan, active users on various social media platforms are about 37 million people out of 76 million (Hayat et al., 2020). A total of 12 universities with a 5-years pharmacy undergraduate program (Pharm-D) and faculty members have been invited to participate in this study via the internet. The current study was completed in two parts, first, the cross-sectional online study was done from August 2019 to March 2020 followed by in-depth interviews of pharmacy faculty members from November 2020 to February 2021. Due to the COVID-19 pandemic, the second phase of the study was delayed. Two phases of mixed methods (quantitative and qualitative) study designs were used to collect the data through online platforms. The first phase (quantitative) is comprised of UGPS irrespective of their study year or semester in Pakistani universities along with faculty members in the second phase (qualitative). Mixed methods design has a positive impact to evaluate both sides of the study area (Khan et al., 2021).

### Part-I: Quantitative Study

#### Study Design and Settings

Firstly, a descriptive cross-sectional study was conducted among UGPSs in 12 (public sector: 08 and private sector: 04) universities of Pakistan located at different geographical regions in the four provinces along with AJK (Azad Jammu and Kashmir) through an online survey. The online platforms were used for the present study between August 2019 and March 2020. We have selected an online approach to collect the data subsequently and with direct contact throughout Pakistan (Khan et al., 2021).

Pharmacy students who gave written informed consent to participate, irrespective of their study year were judged eligible. Students of other subjects were considered ineligible. Students in the first 2 years of their degree were referred to as “junior” pharmacy students while those in the third, fourth, and fifth years were referred to as “senior” pharmacy students.

#### Study Tools

A systematic literature review was conducted to develop the questionnaire for this survey. A questionnaire was developed from the already available literature. A WHO-based questionnaire has been adopted in this study such as; multi-country public awareness survey and Antibiotic Resistance tools previously used by other researchers (Rajiah et al., 2015; Sakeena M. et al., 2018; Hayat et al., 2021). Following the initial survey structure, specialists in pharmacy practice studies (two academicians and one biostatistician) checked the content and face validity of the instrument. Changes were made following the experts’ recommendations for a better understanding of undergraduate students.

Finally, a 50-item questionnaire with 6 sections was developed for the final survey execution. The first section of the questionnaire included questions about the student’s demographics, such as gender, age, study year/semester, university name, and place. To determine the knowledge and use of antibiotics among the students, 8 questions related to ABR knowledge using different options were included. The third section of the questionnaire having 7 questions is about ABR and nonprescription antibiotics. The fourth section was graded on “true/false” with a total of 08 questions. A five-point Likert scale (strongly agree to strongly disagree) was used for sections five and 15 questions were asked with concerns centered on the ABR, a serious public health issue, and antibiotic misuse is a prime factor towards ABR. The last section is related to the pharmacy curriculum which is presently in practice throughout Pakistan at the undergraduate level. A total of 7 questions were included in this last section to understand students’ responses accordingly with the Likert scale (Supplementary File S2).

#### Sampling Technique, Size, and Data Collection

A snowball and convenient sampling technique were used for this study. A total of 414 pharmacy students from the first year to the last year were included.

The questionnaire was created as an online Google form (https://docs.google.com/forms/) with different sections with the study’s objectives listed on the first page. Special monographs related to the safe use of antibiotics were pasted on every page. Various social media sites such as “WhatsApp,” “Facebook Messenger,” and google form links were used to conduct the survey depending on the preferences and convenience of the participants. To encourage participation, participants were given the option of answering all of the questions by simply clicking on a link. All of the data were collected through a Google form, which was then converted into a data analysis software.

#### Data Analysis

The collected data was gathered and analyzed through Statistical Package for Social Sciences (SPSS Version 22: Chicago, IL, United States). Various statistical tools and techniques were used based on the nature of the data. Descriptive statistics were used, frequencies and percentages were obtained and presented in tabular form. Chi-square tests were used for the association of different variables while Mann Whitney and Kruskal Wallis statistical tests were applied for the median difference in the antibiotics, ABR, knowledge, awareness, and current syllabus. Median scores were determined and presented in a table with median and interquartile range (IQR: defines the middle 50% of values from “lowest to highest” when ordered). A *p*-value was considered statistically significant with less than 0.05.

### Part-II: Qualitative Study

The second phase of the study comprised detailed in-depth interviews and an interview guide was developed based on the exploratory and qualitative study design. Faculty members from pharmacy departments were invited for online interviews and advanced emails have been sent before the interview sessions. Detailed interviews were conducted through online WhatsApp voice calls to avoid personal contact due to the COVID-19 pandemic. The main theme was how to curb the misuse of antibiotics and ABR in Pakistan and the role of universities.

A semi-structured interview tool is a useful approach for addressing the issue of ABR and educating pharmacy students on a timely basis. (Mubuuke et al., 2016; Khan et al., 2021). Exploratory designs have many advantages such as the capability and litheness to yield an in-depth observation and investigation of teaching experiences, knowledge, and purpose of the participants to a certain topic (Boyce and Neale, 2006; Tsang et al., 2017). Faculty members/teachers can reduce the number of changes in the knowledge gap regarding ABR and ASM programs in Pakistan. For validation purposes, pilot interviews were carried out and were not included in the final analysis. The interview guide was finalized after the expert’s recommendations, minor changes have been made. The guide includes themes, subthemes, questions with sub-sections in the final version of the interview guide (Supplementary File S2).

Before the interviews, WhatsApp groups were created, and invitations were sent to the faculty members individually. After acceptance of the request for qualitative interviews through WhatsApp voice calls, in-depth online interviews were conducted. The main focus was on education, teaching, pharmacy syllabus, ABR, and the implementation of AMS programs in Pakistan. English or Urdu (national language) was allowed as a medium for response recording and participants were permitted to choose the language of their ease. The individual interview time duration has lasted about 15–50 min. An online voice recorder was used for the recording and the data were stored for further analysis.

Qualitative data analysis was performed and the interviews were analyzed through general inductive thematic analysis (Saqib et al., 2018; Kiger and Varpio, 2020). All recorded data interviews were played back sensibly, and explanations were carefully noted. Then, from the audio recordings, the interviews were verbatim transcribed. Codes were given after the main themes and subthemes were finalized. The saturation point was noted and no other themes were developed once the saturation point was reached (Saunders et al., 2018). Thus, after the 20th interview, further interview discussion was stopped. Lastly, for the further endorsement of the saturation point, six interviews were carried out, and then the analysis was completed accordingly.

## Results

### Part-I (Quantitative)

#### Participants Demographics

A total of 414 UGPS (from 12 universities) have filled the online questionnaire. The majority of the participants were males (*n* = 223, 53.8%) with the age group 19–23 (*n* = 295, 71.2%). Third professional year students have responded the most (*n* = 116, 28.0%) and the student’s participation from public and private sector universities were about (*n* = 289, 69.8% and *n* = 125, 30.1%) respectively (Table 1). The majority of students have taken any type of antibiotics during the last month (*n* = 154, 37.1%) and (*n* = 44, 10.6%) used previously-stored antibiotics.

**TABLE 1:** Undergraduate students demographics characteristics (*n* = 414).

Parameters	N (%)
Gender	
Male	223 (53.8%)
Female	191 (46.1%)
Age Groups (years)	
19–23	295 (71.2%)
24–29	119 (28.7%)
Study Year*	
First Year	65 (15.7%)
Second Year	84 (20.2%)
Third Year	116 (28.0%)
Fourth Year	63 (15.2%)
Fifth Year	86 (20.7%)
University Type	
Public Universities (PU = 08)	289 (69.9%)
Private Universities (PTU = 04)	125 (30.1%)
When did you last take antibiotics?	
Last month	154 (37.1%)
During the past 6 months	132 (31.8%)
During last year	48 (11.5%)
More than a year	80 (19.3.4%)
Where did you get the antibiotics?	
Pharmacy (Drug outlet)	305 (73.6%)
I had saved previously	44 (10.6%)
Friends/Family	64 (15.4%)

*Most of the universities have a semester-based system, so overall year group followed as per the semester.

### Exploring the Antibiotics Knowledge

The majority of participants have good knowledge about antibiotics use and most of them purchased antibiotics through prescription (*n* = 277, 54.8%) during the last month and preferred to obtain them from a Pharmacy. They believe that antibiotics are still available easily and can be obtained (*n* = 210, 50.70%) without prescription, but most of the participants believe in the storage of antibiotics (*n* = 200, 48.30%) at home which is not appropriate. Participants responded to the completion of the therapy, however, students (*n* = 152, 36.70%) stopped taking antibiotics after feeling good with two or three doses. Overall, the students’ knowledge about antibiotics use was correct at a rate of 47%, and less than required knowledge was observed (Table 2).

**TABLE 2:** Exploring antibiotics knowledge and antibiotic use.

Questions	Yes	No	DK*	CR*	Median (IQR)
	N (%)	N (%)	N (%)	N (%)	
Did you get the antibiotics through a prescription during last month?	277 (66.9%)	115 (27.8%)	72 (17.4	277 (66.9	1 (1)
Did you get advice from a Physician or Pharmacist on how to take the antibiotics?	196 (47.30%)	151 (36.5%)	67 (16.2%)	196 (47.30	2 (1)
Is it appropriate, to buy the same antibiotics, or request these from a doctor, if you are sick and they helped you get better when you had the same symptoms before?	109 (26.30%)	265 (64.0%)	40 (9.70	265 (64.0	2 (1)
Do you think this statement is appropriate? to use antibiotics that were given to a friend or family member, as long as they were used to treat the same illness	112 (27.10%)	246 (59.40%)	56 (13.50%)	246 (59.40%)	2 (1)
Antibiotics are the only medicines to treat illness other than infectious diseases	110 (26.50%)	280 (67.60%)	24 (5.8%)	280 (67.60	1 (1)
*Ceftriaxone is first the generation cephalosporin antibiotic class	168 (40.50%)	180 (43.50%)	66 (16.0	180 (43.50%)	1 (0)
Antibiotics are easy to access and can be obtained without a prescription	210 (50.70%)	84 (20.20%)	120 (29.1%)	84 (20.20%)	1 (1)
Prescription is required for antibiotics dispensing at a community pharmacy	198 (47.80%)	87 (21.01%)	129 (31.1%)	198 (47.80%)	2 (0)
Common cold, sore throat, and other flue conditions can be treated with antibiotics	150 (36.20%)	167 (40.3%)	97 (23.5%)	167 (40.3%)	2 (1)
The storage of unnecessary antibiotics at home is good practice and can be stored to treat future illnesses	200 (48.30%)	169 (40.8%)	45 (10.8%)	169 (40.8%)	1 (1)
What do you think, after feeling good, antibiotics should be stopped even after two or three doses?	152 (36.70%)	236 (57.0%)	26 (6.30%)	236 (57.0%)	2 (1)
Antibiotics are widely used in agriculture, and it is one of the reasons for antibiotic resistance	119 (28.70%)	212 (51.20%)	83 (20.0%)	119 (28.70%)	2 (0)

*DK, don’t know; CR, correct rate (*answer: Ceftriaxone is a third generation cephalosporin).

Table 5 has three main sections including; 1) rational use of antibiotics, 2) ABR issue, and current pharmacy curriculum in Pakistan.

#### Rational Use of Antibiotics

The majority of the respondents have strongly agreed (65.2%) that the people must use only prescribed (Median = 1, IQR = 1) antibiotics. Most of the participants strongly agreed that (70.8%) physicians must avoid unnecessary antibiotics prescriptions (Median = 2, IQR = 1). The UGPS answer to prescription-only (*n* = 270, 65.2%) antibiotics was affirmative, indicating that a high percentage of students believed in the sensible use of antibiotics. Most of the participants strongly agreed to stop household storage of unnecessary 183 (44.2%) antibiotics and also the new antibiotics need time to overcome the ABR issue (Med = 2, IQR = 1) Table 5.

#### ABR Issue

The majority of the UGPSs believed in the ABR issue as 173 (41.8%) students agreed to the statement related to ABR as a major issue (Median = 2, IQR = 1). Responsible use of antibiotics 161 (38.9%) may reduce the ABR risk and the rational 231 (55.8%) use of antibiotics will minimize (Med = 2, IQR = 1) the risk of ABR (Table 5).

#### The Current Undergraduate Curriculum of Pharmacy

Overall, the majority of the students (*n* = 198, 47.8%) believe that the current pharmacy syllabus must be updated in good time with the new subjects related to ABR and AMS in Pakistan. The current pharmacy syllabus does not cover (*n* = 133, 32.1%; Median = 3, IQR = 1) the main aspects of ABR. The pharmacy syllabus must include the subjects related to AMR/ABR, AMS, and epidemiology. The most important issue facing by undergraduate students is “Academia and Hospital linkage” which is the need of the hour. During undergraduate studies, only final year students attended hospital internships (*n* = 220, 53.1%; Median = 1, IQR = 2). The hospital internship should focus on AMR/ABR (Table 5).

The private sectors universities students have good knowledge of antibiotics and the related terminologies as compared to public sector universities (Med = 2, IQR = 0; and Med = 1, IQR = 1, *p* < 0.001). The age group (19–23 years) of undergraduate students have good knowledge as compared to the age group (24–29 years) and significant association was found (Median = 2, IQR = 1; and Median = 2, IQR = 0, *p* < 0.05) Table 6.

#### Exploring Knowledge Related to Terminologies and Information Sources

The majority of the participants obtained information from social media (Facebook, WhatsApp, Instagram) regarding different terminologies as shown in Table 3.

**TABLE 3:** Exploring knowledge related to terminologies awareness and their sources.

Questions	PH*	HCW*	FM*	MD*	UGS*	SM*	DN*
Where did you hear about the term: ‘Antimicrobial resistance’?	80 (19.5%)	22 (5.3%)	10 (2.4%)	9 (2.2%)	141 (34.1%)	145 (35.0%)	07 (1.7%)
Where did you hear about the term: ‘Antibiotic Resistance’?	89 (21.5%)	25 (6.0%)	22 (5.3%)	13 (3.10%)	103 (24.8%)	151 (36.5%)	11 (2.7%)
Where did you hear about the term: ‘Superbugs’?	101 (24.3%)	68 (17.8%)	20 (4.8%)	30 (7.2%)	60 (14.4%)	50 (12.0%)	88 (19.8%)
Where did you hear about the term: ‘Antimicrobial stewardship’?	181 (43.7%)	22 (5.3%)	10 (2.4%)	7 (1.7%)	50 (12.0%)	108 (26.1%)	36 (8.69%)
Where did you hear about the term: ‘Drug Resistance’?	117 (28.3%)	20 (4.8%)	05 (1.2%)	05 (1.2%)	119 (28.8%)	142 (34.3%)	06 (1.4%)
Where did you hear about the term: ‘Multidrug Resistance?	81 (19.6%)	21 (5.1%)	8 (1.9%)	11 (2.7%)	127 (30.7%)	165 (39.9%)	01 (0.2%)
Where did you hear about the term: ‘Extensively drug Resistance?	50 (12.0%)	30 (7.2%)	05 (1.2%)	06 (1.4%)	70 (16.9%)	87 (21.0%)	166 (40.0%)
Where did you hear about the: use of antibiotics with caution?	118 (28.5%)	65 (15.7%)	15 (3.6%)	20 (4.8%)	69 (16.6%)	120 (28.9%)	07 (1.7%)
Where did you hear about the: Antibiotic resistance spreading very fast?	98 (23.6%)	64 (15.6%)	25 (6.0%)	10 (2.4%)	85 (20.5%)	117 (28.2%)	15 (3.6%)
Where did you hear about the: national action plan for antimicrobial resiastnce in Pakistan?	90 (21.7%)	15 (3.6%)	05 (1.2%)	20 (4.8%)	30 (7.2%)	150 (36.2%)	104 (25.1%)

*PH, Pharmacist; HCW, Healthcare workers; FM, Family and Friends; MD, Media (print/electronic); UGS, Undergraduate studies; SM, social media (Facebook, WhatsApp, Instagram); DN, don’t know.

**FIGURE 1:**
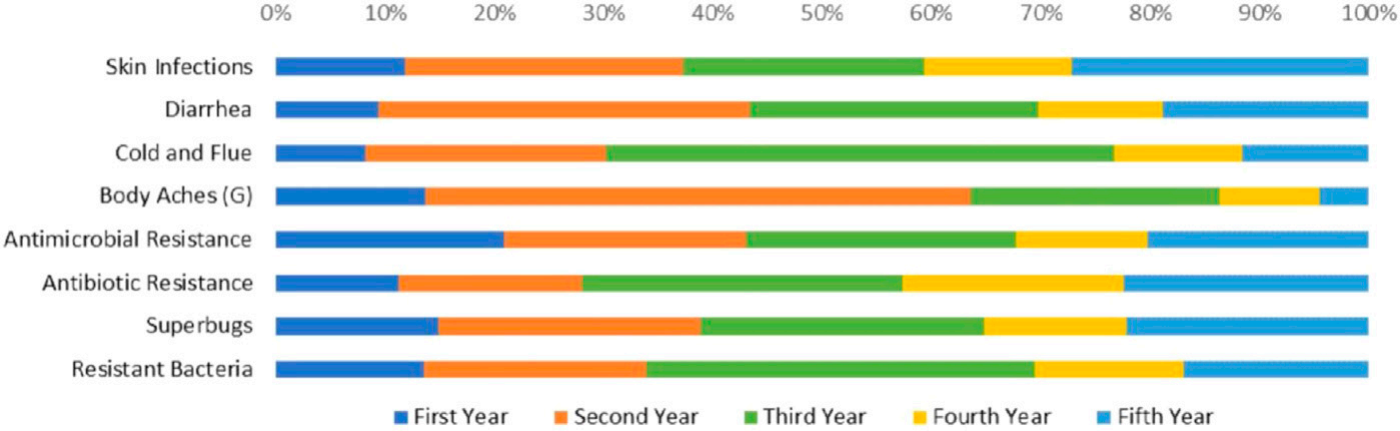
Responses of UGPSs towards antibiotics use in different conditions (skin infections, diarrhea, general body aches) and ABR related terminologies (AMR, ABR, superbugs and resistant bacteria).

Most of the students have heard the terminologies AMR, ABR, or MDR (35, 36.5, 39.9%), and others were studied during the lecture. The AMS-related information was obtained from the pharmacists (*n* = 181, 43.7%) around in practice. Social media usage has increased during the last decade and most academics use social media as a medium for their lectures or sessions, and that is a major reason for how students received information regarding Pakistan AMR-national action plan (*n* = 150, 36.2%). On the other hand, undergraduate students are unaware (*n* = 104, 25.1%) of a national action plan of AMR and the detail is presented in Table 3. The overall study year-wise understanding of different terminologies and different conditions treatment with antibiotics is presented in Figure 1.

#### The Antibiotic Resistance Level of Understanding

Overall, respondents have a good understanding of the ABR, and the majority responded with (54.1%) correct answers. The ABR is a global issue and only (73, 17.6%; *p* > 0.05) responded that it is a worldwide issue including Pakistan. The question of whether use of antibiotics regularly without consultation of physicians or pharmacists can lead to ABR was wrongly answered (162, 39.1%; *p* > 0.05). Only 35 (8.5%; *p* < 0.05) participants did not give the correct answer for the ABR definition as enlisted in Table 4.

**TABLE 4:** Exploring antibiotic resistance knowledge and antibiotic use.

Questions	True	False	Don’t know	Correct rate	Median (IQR)	*p*-Value*
	N (%)	N (%)	N (%)	N (%)		
Antibiotic resistance occurs, when your body becomes resistant to antibiotics, and they no longer work?	335 (80.9%)	35 (8.5%)	44 (10.6%)	335 (80.9%)	1 (0)	0.001
Many infections are becoming increasingly resistant and bacteria to treatment by antibiotics?	303 (73.20%)	83 (20.0%)	28 (6.80%)	303 (73.20%)	1 (0)	0.024
If bacteria are resistant to antibiotics, it can be very difficult or impossible to treat the infections?	35 (8.50%)	342 (82.6%)	37 (8.90%)	342 (82.6%)	2 (0)	0.000
Antibiotic resistance is an issue that could affect me or my family	204 (49.30%)	181 (43.7%)	29 (7.0%)	204 (49.30%)	2 (1)	0.000
Antibiotic resistance is an issue in other countries but not here?	284 (68.60%)	73 (17.6%)	57 (13.8%)	73 (17.6%)	1 (1)	0.092
Antibiotic resistance is only a problem for people who take antibiotics regularly without consultation with a physician?	145 (35.60%)	162 (39.1%)	160 (25.8%)	145 (35.60%)	2 (2)	0.085
Bacteria that are resistant to antibiotics can be spread from person to person?	258 (62.3%)	59 (14.3%)	97 (23.4%)	258 (62.23%)	1 (1)	0.000
Antibiotic-resistant infections could lead medical procedures like surgery, organ transplants, and cancer treatment much more difficult?	134 (32.4%)	208 (50.2%)	72 (17.4%)	134 (32.4%)	2 (1)	0.052

*Pearson Chi-Square Association.

The majority of the participants agreed, (*n* = 173, 41.8%) and strongly agreed, (*n* = 155, 37.4%) that ABR is one of the biggest issues and needs attention. Everyone has a responsibility to use antibiotics wisely and the collective efforts of all the stakeholders can minimize the inappropriate use of antibiotics (Median = 2, IQR = 1) Table 5.

**TABLE 5:** Exploring antibiotics knowledge and antibiotic use.

Questions	Agree		U/N (%)	Disagree		Median (IQR)
	S-A (%)	A (%)		D-A (%)	S-DA (%)	
Rational use of antibiotics						
People should use antibiotics only when they are prescribed by a physician	270 (65.2)	87 (21)	24 (5.8)	12 (2.9)	21 (5.1)	1 (1)
Farmers should give fewer antibiotics to food-producing animals	80 (19.3)	128 (30.9)	115 (27.8)	52 (12.6)	39 (9.4)	2 (1)
People should not keep antibiotics at home and use them later for other illnesses	183 (44.2)	134 (32.4)	39 (9.4)	47 (11.4)	9 (2.2)	2 (1)
Parents should make sure all of their children’s vaccinations are up to date	286 (69.1)	105 (25.4)	13 (3.1)	6 (1.4)	4 (1.0)	1 (1)
People should wash their hands regularly	308 (74.4)	79 (19.4)	13 (3.1)	10 (2.4)	4 (1.0)	1 (1)
Doctors should only prescribe antibiotics when a patient needs them	293 (70.8)	85 (20.5)	7 (1.7)	19 (4.6)	10 (2.4)	2 (1)
Governments should reward the development of new antibiotics with the facilities of drug discovery labs	204 (49.3)	182 (44.0)	20 (4.8)	7 (1.7)	1 (0.2)	1 (1)
Pharmaceutical companies should develop new antibiotics to overcome the ABR issue	205 (68.4)	174 (25.6)	25 (5.6)	6 (1.4)	4 (1.0)	2 (1)
Antibiotic Resistance (ABR) Issue						
Antibiotic resistance is one of the biggest problems the world faces	155 (37.4)	173 (41.8)	68 (16.4)	8 (1.9)	10 (2.4)	2 (1)
Medical experts will solve the problem of antibiotic resistance before it becomes too serious	121 (29.2)	182 (44.0)	78 (18.8)	25 (6.0)	8 (1.9)	2 (2)
Everyone needs to take responsibility for using antibiotics responsibly	161 (38.9)	179 (43.2)	57 (13.8)	17 (4.1)	0 (0.0)	2 (1)
Collective efforts of all the healthcare workers can take part in antibiotic resistance awareness	121 (29.2)	182 (44.0)	78 (18.8)	25 (6.0)	8 (1.9)	2 (1)
I am worried about the impact that antibiotic resistance will have on my health and that of my family	82 (19.8)	248 (59.9)	52 (12.6)	26 (6.3)	6 (1.7)	2 (0)
I am not at risk of getting an antibiotic-resistant infection, as long as I take my antibiotic correctly	77 (18.6)	231 (55.8)	54 (13.0)	42 (10.1)	8 (1.9)	2 (1)
Current undergraduate (Pharm-D) curriculum of Pharmacy						
The ABR-related subjects at the Pharm-D level need time to know the updated and real scenario of resistance	115 (27.8)	198 (47.8)	57 (14.0)	25 (6.0)	18 (4.3)	2 (1)
The pharmacy syllabus provides adequate education regarding antibiotic use and resistance	55 (13.3)	175 (42.3)	163 (39.4)	16 (3.9)	3 (0.7)	2 (1)
One of the reasons for the increase in ABR is the current curriculum as academia can play a role in the ABR issue	121 (29.2)	187 (45.2)	69 (39.4)	31 (7.5)	6 (1.4)	2 (2)
Do you think the current Pharm-D syllabus is old and to be revised immediately?	98 (23.7)	153 (37.0)	112 (27.1)	41 (9.9)	10 (2.4)	2 (1)
Does the current Pharm-D syllabus cover all the aspects of AMR/ABR?	66 (15.9)	133 (32.1)	118 (28.5)	76 (18.4)	21 (5.1)	3 (1)
The current syllabus doesn’t include enough subjects that should cover the AMR/ABR	27 (6.5)	95 (22.9)	160 (38.6)	110 (26.7)	22 (5.3)	3 (2)
Academia and Hospital linkage is the need of time, during Pharm-D internship more focus should be on AMR/ABR	220 (53.1)	74 (17.9)	120 (29.0)	0 (0.0)	0 (0.0)	1 (2)

S-A=strongly agree; A=agree; D-A=disagree; S-DA=strongly disagree.

ABR understanding of students gender-wise was found to be significant (female: Median = 1, IQR = 0; male: Median = 1, IQR = 0, *p* < 0.05) Table 6.

**TABLE 6:** Median scores of related antibiotics knowledge, antibiotics use, awareness of terminologies, ABR, and pharmacy syllabus.

Variables	Median (IQR) ABU	*p*-Value	Median (IQR) K-ABU	*p*-Value	Median (IQR) AW	*p*-Value	Median (IQR) ABR	*p*-Value	Median (IQR) PHC	*p*-Value
Gender										
Male	2 (1)	0.320	2 (1)	0.878	1 (0)	0.129	1 (0)	0.04	2 (2)	0.72
Female	1 (1)		2 (1)		2 (0)		2 (0)		2 (1)	
Age										
19–23	1 (1)	0.721	2 (1)	0.030	1 (0)	0.199	1 (0)	0.57	2 (2)	0.52
24–29	1 (1)		2 (0)		2 (0)		1 (0)		2 (1)	
University Type										
PSUs	1 (0)	0.309	1 (1)	<0.001	1 (0)	0.470	2 (0)	0.57	1 (0)	0.41
PTSUs	2 (0)		2 (0)		2 (0)		2 (0)		2 (0)	

*ABU, antibiotics use; K-ABU, antibiotics knowledge; AW, awareness of terminologies; PHC, pharmacy syllabus.

#### Junior UGPSs Perspectives Towards Antibiotics Use, ABR, AMS, and Pharmacy Curriculum

First and second-year UGPSs were considered junior students. Overall, the junior UGPSs perspectives were low to moderate in terms of antibiotics use, ABR, and AMS. Junior UGPSs have responded to the antibiotics usage directions and stop it (*n* = 75) after getting well. The correct responses were small in number. First-year (*n* = 30) UGPSs were given false answers for the sharing of antibiotics with friends and family and the second-year (*n* = 45) students have given the right answer. Statement related to the ABR, that antibiotics become ineffective after the resistance development and out of 150 junior UGPS 109 answered with the true response. Junior UGPSs have very low and limited knowledge regarding the ABR issue and the majority of the junior UGPSs (*n* = 109) believe that ABR is not an issue here in Pakistan. The junior UGPSs strongly agreed to (*n* = 37) the inclusion of AMR-related subjects in the pharmacy syllabus.

#### Senior UGPSs Perspectives Towards Antibiotics Use, ABR, AMS, and Pharmacy Curriculum

Students from third to a final year were considered senior students. Senior UGPSs have good knowledge regarding the ABR, AMS, and pharmacy syllabus. Senior UGPSs have better responded (*n* = 178) to the antibiotics directions statement and are believed to take antibiotics as directed by the physician. The sharing of antibiotics with family members and friends is one of the causes for ABR and senior UGPSs (*n* = 177) have responded with the true answer. Antibiotics lose their effectiveness once experienced with a resistance pattern, and become ineffective for the bacteria, and senior UGPSs (*n* = 226) reported this with the correct answer. The senior UGPSs also have limited knowledge related to the ABR issue around the globe and a small (*n* = 52) number have answered correctly. Senior UGPSs were strongly agreed to (*n* = 78) the inclusion of AMR-related courses in the pharmacy curriculum in Pakistan.

In the comparison between junior and senior UGPSs with respect to their year, it was found that the senior students have more awareness than a junior in terms of antibiotics use, ABR, AMS, and pharmacy syllabus.

### Part-II (Qualitative)

Overall, 30 faculty members were invited for an online interview through email, and 25 pharmacy faculty members have accepted our invitation. Finally, 20 participants (associate professors = 2, assistant professors = 3, sr. lecturers = 6, and lecturers = 9) were interviewed online with a mean duration of 15 min. Among the total participants, 14 were males with a mean age of 32.5 years and mean experiences of 4.6 years. The thematic analysis of the interviews revealed a total of 7 themes, subthemes 8, and 16 categories enlisted in Table 7.

**TABLE 7:** Detailed summary of themes, sub-themes, and questions for faculty members of.

Question	Main theme	Subthemes
Antimicrobial/antibiotic resistance, one of the major public health issues around the world	The main factors are involved in general and Pakistan suffering largely	**-------------------**
Role of pharmacy students/future pharmacists in ABR and AMSP	Pharmacy graduates or current students have an important role in the reduction of ABR and increasing the rational use of antibiotics	**-------------------**
Specific training regarding ABR and AMSP.	Training on ABR and ASP is mandatory at the institution level for Pharmacy students	Training, symposiums, conferences, and seminars can help to highlight the ABR issue
Courses related to ABR and AMSP	The present Pharmacy syllabus is lacking in courses related to ABR and ASP.	The AMR-related subjects at the Pharm-D level are enough or need to be timely updated
		Students must know about the real scenario of AMR
Pharmacy curriculum currently in practice in Pakistan	The pharmacy curriculum is not up to date regarding the ABR issue	The pharmacy syllabus provides adequate education regarding antibiotic use and resistance
		Do you think the current Pharm-D syllabus is old and to be revised immediately?
		Does the current Pharm-D syllabus cover all the aspects of antimicrobial resistance?
		The current syllabus does not include enough subjects that should cover the AMR
Education and training updated information on AMR/ABR	Pharmacy institutions are responsible to provide adequate training on ABR	Do the Pharmacy institutes provide adequate training on surveillance and up-to-date knowledge for drug-resistant organisms?
		Academia and Hospital linkage is the need of the time, during Pharm-D internship more focus should be on AMR/ABR.
Antimicrobial/Antibiotic Stewardship Programs (ASP)	Antimicrobial/Antibiotic Stewardship Program (ASP) and faculty members role	How can faculty members/teachers of Pharmacy institutions play a role in the ASP implementation?
		Academia and hospital linkage will reduce the gap in Pakistan
		Barriers in the ASP implementation in Pakistani hospitals
		Recommendations for the implementation of ASP activities

The pharmacy faculty members have emphasized the ABR issue and urge practical steps towards ABR awareness among students. Pieces of training are key for the students at the undergraduate level to promote rational antibiotics use. The current pharmacy syllabus must be updated accordingly, and courses related to AMS need to be included. These efforts will lead to a decrease in the AMR and the detailed quotations of the interviews are discussed below.

#### Antimicrobial/Antibiotic Resistance is a Major Public Health Issue

Presently, AMR/ABR is one of the most attenable issues around the globe and more specifically Pakistan needs much attention. The majority of the pharmacy faculty members (P-FM) have mostly the same worried reaction to the issue of ABR.

“Yes, this is one issue being ignored in Pakistan as the world is responding to the ABR issue. ABR will be the next pandemic and we must worry to think about it.” (P-FM: 02).

“The fact is, who will take responsibility to curb the ABR issue? As teachers we can educate our students.” (P-FM: 11).

#### Role of Pharmacy Students in ABR to Promote AMS Programs

Most of the faculty members have emphasized that the students play their role in the ABR and promote the safe and rational use of antibiotics. Pharmacy students are future pharmacists and they should be aware of the ABR issue during their studies. All the efforts at the undergraduate level will lead to the safe use of antibiotics and better clinical outcomes.

“We should prepare and educate our undergraduate students to play a positive role regarding ABR in future.” (P-FM: 05).

#### Training

Training, seminars, symposiums, and conferences regarding ABR and for the promotion and awareness of AMSP is a current need in Pakistan. Universities can play their role in the conducting of such activities and faculty can support such events for the better guidance of students.

“Several symposiums must be conducted by a highly devoted and highly skilled person to promote appropriate use of antibiotics. Another group of individuals is needed to organize and conduct such seminars, not only for students but also for healthcare stakeholders, to ensure proper antibiotic use and education.” (P-FM: 01).

“Different conferences are very important for the AMSP awareness.” (P-FM: 15).

#### Current Pharmacy Syllabus

The current pharmacy syllabus has a deficient of adequate topics and information related to the ABR issue. Moreover, no particular course related to AMS is available at the moment. The majority of the participants urge the immediate up-gradation of the pharmacy curriculum with advanced subjects.

“I strongly advise that the current Pharm-D curriculum must be revised. For a better understanding of rational antibiotic use, AMSP should be incorporated in a significant proportion. To prepare students for future concerns, I am also in favor of expanding the forensic pharmacy courses of the existing syllabus.” (P-FM: 01).

The syllabus has information and topics related to AMR/ABR but it lacks up-to-date information to be provided to the students.

“This is quite accurate that the current syllabus is old, but it needs to be updated. Like the addition of subjects related to the drug resistance and ABR issue must be included in the syllabus.” (P-FM: 09).

#### Courses Related to ABR and AMS

“We have some basic topics in the current syllabus regarding AMR/ABR under the subject of Microbiology but we don’t have a complete subject regarding ABR, so as per my experience a complete subject must be included which could cover the AMR/ABR and about AMS.” (P-FM: 12).

“Subjects related to ABR should be the part of Pharmacy syllabus.” (P-FM: 08).

#### Education and Updated Information on AMR/ABR

Pharmacy faculty members should be aware of the current scenario of ABR. Hands-on training is important for the teachers to be aware of their students and make them prepare to promote the safe use of antibiotics.

“Yes, we have a large number of subjects, with a greater emphasis on pharmaceutics. I believe that teachers require practical training for ABR and AMS.” (P-FM: 05).

Pharmacy institutes do not conduct surveillance for the ABR in their locality and neither get any updates from nearby hospitals about the ABR prevalence.

“No, proper surveillance is not in my notice, I will try to get such information” (P-FM: 03).

“There is no accurate surveillance system exists at pharmacy institution level to provide a current scenario of the AMR.” (P-FM: 01).

The academia and hospital linkage is very important for the pharmacy institutes and their students to get trained during the internships.

“The academia and hospital linkage is vital for the students during Pharm-D studies, as only theory is not enough, a practical approach must calculate the proper dose and study patient medical records. These efforts will help the students in their career.” (P-FM: 02).

“Yes, at every institutional level, a link between academia and hospitals should be developed.” (P-FM: 11).

#### Antimicrobial/Antibiotic Stewardship Programs

ASP-related knowledge must be provided to faculty members as half of the faculty members are not fully aware of the ASP. Seven participants explained the ASP and its implementation in a very good manner.

“I am very aware of ASP as it promotes the safe use of AB and to reduce ABR” (P-FM: 09).

“ASP includes a panel of specialists like clinicians, pharmacists, microbiologists, and other experts who might be able to contribute to the safe and effective use of antibiotics.” (P-FM: 01).

There were several recommendations from the faculty members regarding the AMR/ABR issue and the promotion of ASP.

“Monitoring, advertising, and educating the people will help to reduce the ABR.” (P-FM: 18).

“Leadership, surveillance and tracking of community pharmacies for dispensing of antibiotics and the implementation of ASP at the hospital and community levels is the need of the hour.” (P-FM: 08).

## Discussion

The current study is the first of its kind, a mixed (two phases) design study from Pakistan to explore the knowledge of UGPS regarding AB use, ABR, related terminologies, and views on the current pharmacy curriculum in the first phase while the second phase is comprising a qualitative study and detailed interviews with pharmacy faculty members. The 12 Pakistani university students have good to moderate knowledge about antibiotic use and having average information related to ABR with positive views on the current syllabus for the addition of ABR and AMSP-related information. Pharmacy teachers from different organizations around Pakistan have urged the addition of subjects related to ABR and AMSP in the current pharmacy syllabus.

The majority of the UGPS (first-final year) believe that antibiotics are still available easily without prescription as the same findings reported from the Punjab province (Hayat et al., 2021), while as per Pakistan drug laws it has been forbidden to obtain antibiotics without prescription (Ali et al., 2020). Near to the same results from LMICs it is common to purchase antibiotics without prescription in Bangladesh (45.7%), Vietnam (55.2%), and Ghana (36.1%) (Sulis and Gandra, 2021). Mainly, the staff at pharmacy/drug outlets are involved in the dispensing without prescription antibiotics (Hayat et al., 2019b). The UGPS strongly agreed to stop household storage of unnecessary antibiotics which needs awareness in the public as most of the Asian countries have a higher ratio of household use of antibiotics (Sulis and Gandra, 2021). Completion of antibacterial therapy has a direct link with the positive outcomes, however, UGPS need to be aware of when to stop antibiotics treatment as 36.7% wrongly believed when best to stop taking antibiotics. The safe use of antibiotics with the implementation of AMSP can lead to therapy completion in hospitals (Tamma et al., 2021).

Social media has a very important role in providing information as most of the events related to medicines are live on social sites (Facebook, WhatsApp, Instagram) and a majority of the participants obtained information from social media regarding different terminologies (AMR, MDR, DR, and AMS). Antibiotics are frequently sought via social media, institutional events will quickly intensify antimicrobial resistance debates on social media, and societal participation in the antimicrobial resistance agenda must be sustained (Dyar et al., 2014; Thompson et al., 2021). During the last decade, social media use has risen, and most scholars now use it to deliver seminars or lecture sessions (Askell-Williams et al., 2013) and that is the reason the majority of the students received information (36.2%) regarding Pakistan’s AMR-national action plan through social media.

More than half of UGPS responded with (54.1%) correct answers related to ABR and it is recommended that inappropriate use of antibiotics could potentiate the risk of ABR as reported by HL Wilson and his colleagues (Wilson et al., 2019). Apart from the basic knowledge, 39% of the total UGPS still believed that the regular use of antibiotics without consultation of the physician is accurate. A study from Saudi Arabia indicates that inability to afford a consultation with a physician (65.3%) is the key reason for the direct use of antibiotics (Hadi et al., 2016), while this prevalence is 49.0% in Jordan as they use antibiotics that have been leftover without consultation of a doctor (Shehadeh et al., 2012). The majority of students were agreed and strongly agreed (41.8%, 37.4%) that ABR is one of the major problems of attention. Everyone has to use antibiotics responsibly, and concerted actions from all stakeholders will reduce antibiotic misuse (Khan FU. et al., 2020; Khan et al., 2020c; Khan Z. et al., 2020; Khan et al., 2021).

The present study respondents were familiar with basic knowledge related to ABR as this is part of the PharmD syllabus, but lacking information related to AMS. Overall, the majority of students (47.8%) agreed that the existing pharmacy syllabus in Pakistan needs to be revised as Hayat et al. have focused (Hayat et al., 2021a). The UGPS have believed that the present pharmacy syllabus does not cover all the aspects, subjects relevant to ABR and AMS are additionally necessary for Pakistan as the same report from Sri Lankan universities pharmacy students (Sakeena MHF. et al., 2018). Our previous study has also focused on the implementation of AMS programs at the community level (Khan et al., 2021). Although ABR is now a part of many pharmacy schools’ curricula, knowledge on stewardship activities is still lacking. Antibiotic stewardship services have been widely implemented in the pharmaceutical curricula of many countries, including the United States and South Africa (Kufel et al., 2018; Khan Y. et al., 2020).

Teachers or faculty members have the key role to prepare the future pharmacist to face the challenges particularly the ABR challenge in Pakistan. When it comes to the topic of ABR, the majority of pharmacy faculty members (P-FM) have a similar worrying response as ABR is also being reported as an ethical challenge (Littmann et al., 2015). The majority of faculty members have stressed the importance of students participating in the ABR and promoting the effective and fair use of antibiotics. WHO has been conducted an international survey on the promotion of medicine as an educational initiative for pharmacy and medical students in 110 countries, but more studies are needed specifically on the safe use of antibiotics (Mintzes and Organization, 2005). In Pakistan, training, workshops, symposiums, and conferences on ABR and AMSP promotion and awareness are urgently required as emphasized by the majority of teachers. According to the findings of Atif et al., the government can adopt training courses for pharmacists to improve their performance, and institutional environments should play a role in antibiotic competency and training programs as no such training was offered to new pharmacists (Atif et al., 2020).

In 2004, the Pakistani pharmacy curriculum was upgraded from a four-year Bachelor of Pharmacy (B. Pharm) to a five-year PharmD and then revised in 2011 and again in 2013 (Nazir et al., 2011; Khan and Abbas, 2014). The current pharmacy curriculum lacks appropriate topics and knowledge on the ABR issue. More precisely, there is currently no AMSP-related course available in the present pharmacy syllabus. The majority of participants believed that pharmacy education should be upgraded immediately to include specialized subjects. More work is needed to modernize the current curriculum to meet the international standards (Khan, 2011; Saleem, 2015). The huge knowledge gap among pharmacy undergraduates is an indicator of the curriculum up-gradation (Nazir et al., 2011). The curriculum up-gradation will promote health and wellbeing in the masses (Gajdács et al., 2020) and can reduce the ABR load on the already drooping health care system of the country.

This study revealed different aspects and the understanding of pharmacy students on ABR, AMSP, and teachers of pharmacy institutes highlighted the core issues, especially the present syllabus, which has ABR-related topics but under the scope of microbiology, which is not enough. In addition, academia and hospital linkage is the need of the hour, as to properly understand the idea of ABR, pharmacy students should be allowed to engage in hospital ward rounds. Moreover, the government should introduce antibiotic stewardship services in all Pakistani healthcare settings (Hayat et al., 2019b; Hayat et al., 2021), including a platform for healthcare practitioners and pharmacy students to explore an evidence-based solution to ABR.

The present study has certain limitations; firstly, this study has used a convenience sampling method and universities (public and private sectors) were selected as per student’s availability, which may have resulted in selection bias. Secondly, a limited number of universities were included and public sector universities are included more than the private sector. The results may not be comparable as the education standard of the pharmacy institutions are not the same. Nonetheless, this is a mixed-methods study that provides the most recent insight of pharmacy students related to ABR and AMS programs regardless of their study year and the views of pharmacy teachers are attentional.

## Conclusion

This two-phase research conducted with students and pharmacy teachers from Pakistani universities revealed gaps in knowledge about antibiotic use, antimicrobial resistance, and stewardship initiatives. Nevertheless, students have good knowledge with positive attention towards different approaches used to manage ABR in Pakistan and teachers have urged to promote the safe use of antibiotics. A significant association was found between university type and antibiotics use among the students. Different pieces of training and events related to ABR and AMS along with academia and hospital linkage are needed in Pakistan. ABR and AMS-related subjects are needed to include in the current pharmacy curriculum of Pakistan.

## Data Availability

The raw data supporting the conclusions of this article will be made available by the authors, without undue reservation.

## Abbreviations

ABR, antibiotic resistance; AMS, antimicrobial stewardship; ASP, antibiotic stewardship programs; AMSP, antimicrobial/antibiotic stewardship programs; LMICs, lower middle-income countries; PharmD, Doctor of Pharmacy; UGPS, undergraduate pharmacy students; WHO, World Health Organization.

## References

[B1] Ali M. Abbasi B. H. Ahmad N. Fazal H. Khan J. Ali S. S. (2020). Over-the-counter Medicines in Pakistan: Misuse and Overuse. Lancet 395, 116. 10.1016/S0140-6736(19)32999-X 31929013

[B2] Ashraf M. S. Chung P. Neukirch A. Bergman S. Cavalieri R. J. Ortmeier R. (2020). Impact of Training Consultant Pharmacists on Antimicrobial Stewardship Programs in Long-Term Care Facilities. Infect. Control. Hosp. Epidemiol. 41, s446–s448. 10.1017/ice.2020.1116

[B54] Askell-Williams H. Dix K. L. Lawson M. J. Slee P. T. (2013). Quality of Implementation of a School Mental Health Initiative and Changes Over Time in Students’ Social and Emotional Competencies. Sch. Eff. Sch. Improv. 24, 357–381. 10.1080/09243453.2012.692697

[B3] Atif M. Asghar S. Mushtaq I. Malik I. (2020). Community Pharmacists as Antibiotic Stewards: A Qualitative Study Exploring the Current Status of Antibiotic Stewardship Program in Bahawalpur, Pakistan. J. Infect. Public Health 13, 118–124. 10.1016/j.jiph.2019.07.003 31548165

[B4] Boyce C. Neale P. (2006). Conducting In-Depth Interviews: A Guide for Designing and Conducting In-Depth Interviews for Evaluation Input. Massachusetts, USA: Pathfinder International.

[B5] Burns K. W. Johnson K. M. Pham S. N. Egwuatu N. E. Dumkow L. E. (2020). Implementing Outpatient Antimicrobial Stewardship in a Primary Care Office through Ambulatory Care Pharmacist-Led Audit and Feedback. J. Am. Pharm. Assoc. (2003) 60, e246–e251. 10.1016/j.japh.2020.08.003 32861616

[B6] Carvalho C. M. D. S. (2021). Knowledge and Perceptions of Antibiotic Resistance Stewardship Among Prehealth and Agriculture Students. Minneapolis: Walden University.

[B7] Chokshi A. Sifri Z. Cennimo D. Horng H. (2019). Global Contributors to Antibiotic Resistance. 11 **,** 36. 10.4103/jgid.jgid_110_18 PMC6380099 30814834

[B8] OECD (2019). Stemming the Superbug Tide: Just a Few Dollars More. Paris: OECD Publishing.

[B9] CDC (2017). Antibiotic Use in the United States, 2017: Progress and Opportunities. Atlanta, GA: Centers for Disease Control and Prevention.

[B10] Dellit T. H. Owens R. C. Mcgowan J. E. Gerding D. N. Weinstein R. A. Burke J. P. (2007). Infectious Diseases Society of America and the Society for Healthcare Epidemiology of America Guidelines for Developing an Institutional Program to Enhance Antimicrobial Stewardship. Clin. Infect. Dis. 44, 159–177. 10.1086/510393 17173212

[B11] Dyar O. J. Castro-Sánchez E. Holmes A. H. (2014). What Makes People Talk about Antibiotics on Social media? A Retrospective Analysis of Twitter Use. J. Antimicrob. Chemother. 69, 2568–2572. 10.1093/jac/dku165 24862092 PMC4130384

[B12] Fay L. N. Wolf L. M. Brandt K. L. Deyoung G. R. Anderson A. M. Egwuatu N. E. (2019). Pharmacist-led Antimicrobial Stewardship Program in an Urgent Care Setting. Am. J. Health Syst. Pharm. 76, 175–181. 10.1093/ajhp/zxy023 30689745 PMC6366123

[B13] Founou R. C. Founou L. L. Essack S. Y. (2017). Clinical and Economic Impact of Antibiotic Resistance in Developing Countries: A Systematic Review and Meta-Analysis. PLoS ONE 12, e0189621. 10.1371/journal.pone.0189621 29267306 PMC5739407

[B14] Gajdács M. Paulik E. Szabó A. (2020). Knowledge, Attitude and Practice of Community Pharmacists Regarding Antibiotic Use and Infectious Diseases: a Cross-Sectional Survey in Hungary (KAPPhA-HU). Antibiotics (Basel) 9, 41. 10.3390/antibiotics9020041 PMC7168197 31973119

[B15] García L. P. O. Kaur K. Brand H. Schröder-Bäck P. (2021). Scenario Planning: An Alternative Approach to European Commission for Combating Antimicrobial Resistance by 2050. XVI, SEEJPH, 1–11. 10.11576/seejph-4312

[B16] Goujon A. Wazir A. Gailey N. (2020). Pakistan : un pays de plus de 200 millions d'habitants en retard dans la transition démographique. Popul. Societies N°576, 1–4. 10.3917/popsoc.576.0001

[B17] Hadi M. A. Karami N. A. Al-Muwalid A. S. Al-Otabi A. Al-Subahi E. Bamomen A. (2016). Community Pharmacists' Knowledge, Attitude, and Practices towards Dispensing Antibiotics without Prescription (DAwP): a Cross-Sectional Survey in Makkah Province, Saudi Arabia. Int. J. Infect. Dis. 47, 95–100. 10.1016/j.ijid.2016.06.003 27343987

[B18] Hayat K. Jamshed S. Rosenthal M. Haq N. U. Chang J. Rasool M. F. (2021). Understanding of Pharmacy Students towards Antibiotic Use, Antibiotic Resistance and Antibiotic Stewardship Programs: A Cross-Sectional Study from Punjab, Pakistan. Antibiotics (Basel) 10, 66. 10.3390/antibiotics10010066 33445511 PMC7827071

[B19] Hayat K. Li P. Rosenthal M. Xu S. Chang J. Gillani A. H. (2019a). Perspective of Community Pharmacists about Community-Based Antimicrobial Stewardship Programs. A Multicenter Cross-Sectional Study from China. Expert Rev. Anti Infect. Ther. 17, 1043–1050. 10.1080/14787210.2019.1692655 31714841

[B20] Hayat K. Rosenthal M. Gillani A. H. Zhai P. Aziz M. M. Ji W. (2019b). Perspective of Pakistani Physicians towards Hospital Antimicrobial Stewardship Programs: a Multisite Exploratory Qualitative Study. Int. J. Environ. Res. Public Health 16, 1565. 10.3390/ijerph16091565 PMC6539566 31060262

[B21] Hayat K. Rosenthal M. Xu S. Arshed M. Li P. Zhai P. (2020). View of Pakistani Residents toward Coronavirus Disease (COVID-19) during a Rapid Outbreak: A Rapid Online Survey. Int. J. Environ. Res. Public Health 17, 3347. 10.3390/ijerph17103347 PMC7277197 32408528

[B22] Hofer U. (2019). The Cost of Antimicrobial Resistance. Nat. Rev. Microbiol. 17, 3. 10.1038/s41579-018-0125-x 30467331

[B23] Ingram P. R. Seet J. M. Budgeon C. A. Murray R. (2012). Point-prevalence Study of Inappropriate Antibiotic Use at a Tertiary Australian Hospital. Intern. Med. J. 42, 719–721. 10.1111/j.1445-5994.2012.02809.x 22697156

[B24] Khan F. U. Khan F. U. Hayat K. Ahmad T. Khan A. Chang J. (2021). Knowledge, Attitude, and Practice on Antibiotics and its Resistance: A Two-phase Mixed-Methods Online Study Among Pakistani Community Pharmacists to Promote Rational Antibiotic Use. Int. J. Environ. Res. Public Health 18, 1320. 10.3390/ijerph18031320 33535695 PMC7908617

[B25] Khan F. U. Fang Y. Khan Z. Khan F. U. Malik Z. I. Ahmed N. (2020a). Occurrence, Associated Risk Factors, and Treatment of Surgical Site Infections in Pakistan. Eur. J. Inflamm. 18, 2058739220960547. 10.1177/2058739220960547

[B26] Khan F. U. Khan F. U. Hayat K. Chang J. Saeed A. Khan Z. (2020b). Knowledge, Attitude and Practices Among Consumers toward Antibiotics Use and Antibiotic Resistance in Swat, Khyber-Pakhtunkhwa, Pakistan. Expert Rev. Anti Infect. Ther. 18, 937–946. 10.1080/14787210.2020.1769477 32516001

[B27] Khan F. U. Khan Z. Ahmed N. (2020c). A General Overview of Incidence, Associated Risk Factors, and Treatment Outcomes of Surgical Site Infections. Indian J. Surg. 82, 1–11. 10.1007/s12262-020-02071-8

[B28] Khan N. Abbas A. (2014). The Need to Modernize Clinical Pharmacy Curriculum in Undergraduate Pharmacy Teaching Institutes of Pakistan. Med. Sci. 10 **,** 12–13.

[B29] Khan T. (2011). Challenges to Pharmacy and Pharmacy Practice in Pakistan. Amj 4, 230–235. 10.4066/amj.2011.488

[B30] Khan Y. Kritiotis L. Coetzee R. Mccartney J. Boschmans S. A. (2020d). An Antimicrobial Stewardship Curriculum to Incorporate in the South African Bachelor of Pharmacy Degree Program. Am. J. Pharm. Educ. 84, ajpe7669. 10.5688/ajpe7669 32773825 PMC7405307

[B31] Khan Z. Ahmed N. Rehman A. U. Khan F. U. Saqlain M. Martins M. A. P. (2020e). Audit of Pre-operative Antibiotic Prophylaxis Usage in Elective Surgical Procedures in Two Teaching Hospitals, Islamabad, Pakistan: An Observational Cross-Sectional Study. PloS one 15, e0231188. 10.1371/journal.pone.0231188 32255809 PMC7138312

[B32] Kiger M. E. Varpio L. (2020). Thematic Analysis of Qualitative Data: AMEE Guide No. 131. Med. Teach. 13142, 846–854. 10.1080/0142159X.2020.1755030 32356468

[B33] Koivusalo M. Mackintosh M. (2008). Global Public Health Security: Inequality, Vulnerability and Public Health System Capabilities. Dev. Change 39, 1163–1169. 10.1111/j.1467-7660.2008.00514.x 32313301 PMC7163547

[B34] Kufel W. D. Jeffres M. N. Macdougall C. Cho J. C. Marx A. H. Williams D. M. (2018). Antimicrobial Stewardship Education in US Colleges and Schools of Pharmacy. J. Antimicrob. Chemother. 73, 2252–2258. 10.1093/jac/dky166 29846603

[B35] Littmann J. Buyx A. Cars O. (2015). Antibiotic Resistance: an Ethical challenge. Int. J. Antimicrob. Agents 46, 359–361. 10.1016/j.ijantimicag.2015.06.010 26242553

[B36] Malik U. R. Chang J. Hashmi F. Atif N. Basir H. Hayat K. (2021). A Simulated Client Exploration of Nonprescription Dispensing of Antibiotics at Drugstores for Pediatric Acute Diarrhea and Upper Respiratory Infection in Lahore, Pakistan. Infect. Drug Resist. 14, 1129–1140. 10.2147/IDR.S301812 33790584 PMC7997541

[B37] Martin J. K. Sheehan J. P. Bratton B. P. Moore G. M. Mateus A. Li S. H. (2020). A Dual-Mechanism Antibiotic Kills Gram-Negative Bacteria and Avoids Drug Resistance. Cell 181, 1518–e14. 10.1016/j.cell.2020.05.005 32497502 PMC7780349

[B38] Mintzes B. Organization W. H. (2005). Educational Initiatives for Medical and Pharmacy Students about Drug Promotion: An International Cross-Sectional Survey. Geneva: World Health Organization.

[B39] Mubuuke A. G. Louw A. J. Van Schalkwyk S. (2016). Utilizing Students' Experiences and Opinions of Feedback during Problem Based Learning Tutorials to Develop a Facilitator Feedback Guide: an Exploratory Qualitative Study. BMC Med. Educ. 16, 6–7. 10.1186/s12909-015-0507-y 26753932 PMC4709989

[B40] Nazir M. Naqvi I. I. Khan A. Tahir A. Taha N. Hussain I. (2011). Instruction Manual of Systemic Approach to Teaching and Learning (SATL). Pak. J. Chem. 1, 168–175. 10.15228/2011.v01.i04.p05

[B41] Rajiah K. Ren W. S. Jamshed S. Q. Health P. (2015). Evaluation of the Understanding of Antibiotic Resistance Among Malaysian Pharmacy Students at Public Universities: an Exploratory Study. J. Infect. Public Health 8, 266–273. 10.1016/j.jiph.2014.11.003 25530352

[B42] Sakeena M. H. F. Bennett A. A. Jamshed S. Mohamed F. Herath D. R. Gawarammana I. (2018a). Investigating Knowledge Regarding Antibiotics and Antimicrobial Resistance Among Pharmacy Students in Sri Lankan Universities. BMC Infect. Dis. 18, 209–211. 10.1186/s12879-018-3107-8 29739360 PMC5941408

[B43] Sakeena M. Bennett A. A. Jamshed S. Mohamed F. Herath D. R. Gawarammana I. (2018b). Investigating Knowledge Regarding Antibiotics and Antimicrobial Resistance Among Pharmacy Students in Sri Lankan Universities. BMC Infect. Dis. 18 **,** 1–11. 10.1186/s12879-018-3107-8 29739360 PMC5941408

[B44] Saleem A. J. H. (2015). The Current Needs of Pakistani PharmD Curriculum: A Perspective of Pharmacoeconomics. Pharmacoepidemiol. Clin. Pharm. 7, 1–2. 10.21065/19204159.7.2.106

[B45] Saqib A. Atif M. Ikram R. Riaz F. Abubakar M. Scahill S. (2018). Factors Affecting Patients' Knowledge about Dispensed Medicines: A Qualitative Study of Healthcare Professionals and Patients in Pakistan. PLoS One 13, e0197482. 10.1371/journal.pone.0197482 29856753 PMC5983558

[B46] Sarfraz I. Rasul A. Hussain G. Hussain S. M. Samiullah K. Rasool B. (2020). “Global and Temporal Trends in the Use of Antibiotics and Spread of Antimicrobial Resistance,” in Antibiotics and Antimicrobial Resistance Genes (Berlin: Springer), 81–94. 10.1007/978-3-030-40422-2_4

[B47] Saunders B. Sim J. Kingstone T. Baker S. Waterfield J. Bartlam B. (2018). Saturation in Qualitative Research: Exploring its Conceptualization and Operationalization. Qual. Quant 52, 1893–1907. 10.1007/s11135-017-0574-8 29937585 PMC5993836

[B48] Shehadeh M. Suaifan G. Darwish R. M. Wazaify M. Zaru L. Alja'fari S. (2012). Knowledge, Attitudes and Behavior Regarding Antibiotics Use and Misuse Among Adults in the Community of Jordan. A Pilot Study. Saudi Pharm. J. 20, 125–133. 10.1016/j.jsps.2011.11.005 23960783 PMC3744980

[B49] Shi L. Chang J. Liu X. Zhai P. Hu S. Li P. (2020). Dispensing Antibiotics without a Prescription for Acute Cough Associated with Common Cold at Community Pharmacies in Shenyang, Northeastern china: A Cross-Sectional Study. Antibiotics (Basel) 9, 163. 10.3390/antibiotics9040163 PMC7235837 32268530

[B50] Sulis G. Gandra S. (2021). Access to Antibiotics: Not a Problem in Some LMICs. Lancet Glob. Health 9, e561-e562. 10.1016/s2214-109x(21)00085-1 33713631

[B51] Tamma P. D. Miller M. A. Dullabh P. Ahn R. Speck K. Gao Y. (2021). Association of a Safety Program for Improving Antibiotic Use with Antibiotic Use and Hospital-Onset Clostridioides Difficile Infection Rates Among US Hospitals. JAMA Netw. Open 4, e210235. 10.1001/jamanetworkopen.2021.0235 33635327 PMC7910818

[B52] Thompson W. Emmott R. Barber S. (2021). Antibiotics and Toothache: a Social media Review. Int. J. Pharm. Pract. 29, 210-217. 10.1093/ijpp/riaa024. 33880539

[B53] Tsang S. Royse C. F. Terkawi A. S. (2017). Guidelines for Developing, Translating, and Validating a Questionnaire in Perioperative and Pain Medicine. 11 **,** S80. 10.4103/sja.sja_203_17 PMC5463570 28616007

[B55] Wilson H. L. Daveson K. Del Mar C. B. (2019). Optimal Antimicrobial Duration for Common Bacterial Infections. Aust. Prescr 42, 5–9. 10.18773/austprescr.2019.001 30765902 PMC6370607

