# Supplementary material for: Exploring Undergraduate Pharmacy Students Perspectives Towards Antibiotics Use, Antibiotic Resistance, and Antibiotic Stewardship Programs Along With the Pharmacy Teachers’ Perspectives: A Mixed-Methods Study From Pakistan
Source: Front Pharmacol. 2021 Nov 8;12:754000. doi: 10.3389/fphar.2021.754000 (PMC8606649; doi:10.3389/fphar.2021.754000)
Supplement: Supplementary file 1 [file DataSheet1.pdf]

Participant Information Booklet

The booklet includes:

- Background
- Informed Consent declaration
- Questionnaire (Quantitative)
- Interview Guide (Qualitative)

EXPLORING THE ROLE OF PHARMACY STUDENTS AND TEACHERS IN ANTIBIOTIC RESISTANCE AND ANTIBIOTIC STEWARDSHIP PROGRAMS IN PAKISTAN: TWO PHASES MIXED-METHODS ONLINE STUDY

The ability of bacteria or other organisms to survive the effects of antibiotics is known as antibiotic resistance. Antibiotic resistance occurs when bacteria develop to the point that antibiotics, additives, or other agents used to treat or prevent infections no longer work. Antibiotic stewardship is a systematic approach to improving antibiotic stewardship, patient outcomes, microbial resistance, and the spread of infections caused by multidrug-resistant species.

A. PARTICIPANT INFORMATION SHEET

You are being invited to participate in a research study titled "Exploring The Role Of Pharmacy Students And Teachers In Antibiotic Resistance And Antibiotic Stewardship Programs In Pakistan: Two Phases Mixed-Methods Online Study".

Purpose of study:

This mixed method study aims to assess the understanding of undergraduate pharmacy students towards antibiotic use and antibiotic resistance and antibiotic stewardship programs along with pharmacy faculty members fallowing semi structured interviews.

Your Participation:

Your participation in this study is entirely voluntary and you can withdraw at any time. We believe there are no known risks associated with this research study; however, as with any online related activity, the risk of a breach is always possible. To the best of our ability, your answers in this study will remain confidential. We assure you that all data collected will be anonymous. All data will be stored electronically on password-protected media for a maximum of three years. All data collection, storage, and processing will comply with the principles of the Data Protection.

B. INFORMED CONSENT

You will be asked to sign an informed consent stating that you understand the nature of the study and what is required from you in the study. Participation in this study is voluntary and will not affect your privacy. You are free at any time to change your mind and withdraw from the study without needing to justify your decision.

* By completing this survey please click AGREE to indicate that:

• You are a undergraduate Pharmacy Student

• You are currently studying Pharm-D

• You have read the participant information sheet associated with this study

Sign (optional) _____________

PART 1: QUESTIONAIRE

Section – I:

DEMOGRAPHICS: Circle your answer

S1. What is your age?

1. 20 – 25
2. 26 - 34
3. 35- 44
4. 45+

S2. What is your gender?

1. Male 2. Female

S3. What is your name of the University?

____

S4. What is your year of study at university?

1. 1st year 2. 2nd year 3. 3rd year 4. 4th year 5. 5th Year

Section – II:

Main Questionnaire:

Use of Antibiotics (Circle your Answer)

1) When did you last take antibiotics?

1. In the last month
2. In the last 6 months
3. In the last year
4. More than a year ago
5. Never
6. Can't remember

If 'Never' circle code 5, go straight to Question 5

2) On that occasion, did you get the antibiotics from a doctor's prescription

1. Yes 2. No 3. Can't remember

3) On that occasion, did you get advice from a doctor, nurse or pharmacist on how to take them

1. Yes, I received advice on how to take them (e.g. with food for 7 days)
2. No 3. Can't remember

4) On that occasion, where did you get the antibiotics?

1. Medical store or Pharmacy
2. Stall or hawker
3. The internet
4. Friend or family member
5. I had them saved up from the previous time
6. Somewhere/someone else
7. Can't remember

Section – III:

KNOWLEDGE ABOUT ANTIBIOTICS

5) When do you think you should stop taking antibiotics once you've begun treatment?

1. When you feel better
2. When you've taken all the antibiotics as directed
3. Don't know

6) Do you think this statement is 'true' or 'false?'
"It's okay to use antibiotics that were given to a friend or family member, as long as they were used to treat the same illness"

1. True 2. False 3. Don't know

7) Do you think this statement is 'true' or 'false?'
"It's okay to buy the same antibiotics, or request these from a doctor if you are sick and they helped you get better when you had the same symptoms before"

1. True 2. False 3. Don't know

8) Do you think these conditions can be treated with antibiotics?
You may circle more than one answer

1. HIV/AIDS
2. Gonorrhea
3. Bladder infection or urinary tract infection (UTI)
4. Diarrhea
5. Cold and flu
6. Fever
7. Malaria
8. Measles
9. Skin or wound infection
10. Sore throat
11. Body aches
12. Headaches

KNOWLEDGE ON ANTIBIOTICS RESISTANCE

9) Have you heard any of the following terms? You may circle more than one answer

1. Antibiotic resistance
2. Superbugs
3. Antimicrobial resistance
4. AMR
5. Drug resistance
6. Antibiotic-resistant bacteria

10) You may circle more than one answer for 10.1, 10.2, 10.3, 10.4, 10.5 and 10.6

10.1. If answered Yes in Question 9) to 'Antibiotic resistance'. Where did you hear about the term: 'Antibiotic Resistance'?

1. Doctor or nurse 2. Pharmacist 3. Family member or friend (including on social media)
4. Media (newspaper, TV, Radio) 5. Family Members or Friends
6. Specific Study 7. Undergraduate study 8. Can't remember

10.2. If answered Yes in Question 9) to 'Superbugs'

Where did you hear about the term: 'Superbugs'?

1. Doctor or nurse 2. Pharmacist 3. Family member or friend (including on social media)
4. Media (newspaper, TV, Radio) 5. Family Members or Friends
6. Specific Study 7. Undergraduate study 8. Can't remember

10.3. If answered Yes in Question 9) to 'Antimicrobial resistance'

Where did you hear about the term: 'Antimicrobial resistance'?

1. Doctor or nurse 2. Pharmacist 3. Family member or friend (including on social media)
4. Media (newspaper, TV, Radio) 5. Family Members or Friends
6. Specific Study 7. Undergraduate study 8. Can't remember

10.4. If answered Yes in Question 9) to 'AMR'

Where did you hear about the term: 'AMR'?

1. Doctor or nurse 2. Pharmacist 3. Family member or friend (including on social media)
4. Media (newspaper, TV,Radio) 5. Family Members or Friends
6. Specific Study 7. Undergraduate study 8. Can't remember

10.5. If answered Yes in Question 9) to 'Drug Resistance'

Where did you hear about the term: 'Drug Resistance'?

1. Doctor or nurse 2. Pharmacist 3. Family member or friend (including on social media)
4. Media (newspaper, TV,Radio) 5. Family Members or Friends
6. Specific Study 7. Undergraduate study 8. Can't remember

10.6. If answered Yes in Question 9) to 'Antibiotic- resistant bacteria'

Where did you hear about the term: 'Antibiotic- resistant bacteria'?

1. Doctor or nurse 2. Pharmacist 3. Family member or friend (including on social media)
4. Media (newspaper, TV,Radio) 5. Family Members or Friends
6. Specific Study 7. Undergraduate study 8. Can't remember

Section – IV:

11) Please indicate whether you think the following statements are 'true' or 'false'

	**Statement**	**True**	**False**	**Don't Know**
1	Antibiotic resistance occurs when your body becomes resistant to antibiotics and they no longer work as well			
2	Many infections are becoming increasingly resistant to treatment by antibiotics			
3	If bacteria are resistant to antibiotics, it can be very difficult or impossible to treat the infections they cause			
4	Antibiotic resistance is an issue that could affect me or my family			
5	Antibiotic resistance is an issue in other countries but not here			
6	Antibiotic resistance is only a problem for people who take antibiotics regularly			
7	Bacteria that are resistant to antibiotics can be spread from person to person person			
8	Antibiotic-resistant infections could make medical procedures like surgery, organ transplants and cancer treatment much more difficult.			

Section – V:

12) On the scale down shown, how much do you agree the following actions would help address the problem of antibiotic resistance?

	Statement	**Agree Strongly**	**Agree Slightly**	**Neither agree nor**	**Disagree Slightly**	**Disagree Strongly**
1	People should use antibiotics only when they are prescribed by a doctor					
2	Farmers should give fewer antibiotics to food-producing animals					
3	People should not keep and use them later for other illnesses					
4	Parents should make sure all of their children's vaccinations are up to date					
5	People should wash their hands regularly					
6	Doctors should only prescribe antibiotics when they are needed					
7	Governments should reward the development of new antibiotics					
8	Pharmaceutical companies should develop new antibiotics					

Section – VI:

13) On the scale shown, how much do you agree with the following statement?

	Statement	**Agree Strongly**	**Agree Slightly**	**Neither agree nor disagree**	**Disagree Slightly**	**Disagree Strongly**
1	Antibiotic resistance is one of the biggest problems the world faces					
2	Medical experts will solve the problem of antibiotic resistance before it becomes too serious					
3	Everyone needs to take responsibility for using antibiotics responsibly					
4	There are not much people like me can do to stop antibiotic resistance					
5	I am worried about the impact that antibiotic resistance will have on my health and that of my family					
6	I am not at risk of getting an antibiotic resistant infection, as long as I take my antibiotic correctly					

Section – VII:

USE OF ANTIBIOTICS IN AGRICULTURE

14) Do you think antibiotics are widely used in agriculture, including food-producing animals in your country?

1. Yes 2. No 3. Don't know

15) On the scale shown, how much do you agree with the following statement?

	Statement	**Agree Strongly**	**Agree Slightly**	**Neither agree nor disagree**	**Disagree Slightly**	**Disagree Strongly**
1	The AMR-related subjects at the Pharm-D level is need time to know the updated and real scenario of resistance.					
2	The pharmacy syllabus provides adequate education regarding antibiotic use and resistance					
3	One of the reasons for increase AMR is the current [illegible]					
4	Do you think the current Pharm-D syllabus is old and to be revised immediately					
5	Does the current Pharm-D syllabus cover all the aspects of Antimicrobial resistance?					
6	The current syllabus doesn't include enough subjects that should cover the AMR					
7	The Pharmacy institutes perform adequate surveillance for drug-resistant organism					
8	Academia and Hospital linkage is the need of time, during Pharm-D internship more focus should be on AMR					

PART 2: INTERVIEW GUIDE

Exploring the Role Pharmacy Teachers in Antibiotic Resistance and Antibiotic Stewardship Program in, Pakistan

I. **Demographics:**

1) Name: ____________________
2) Age: ____________________
3) Gender: __________________
4) Name of University: ___________________
5) Degree: ___________________
6) Current Position: _________________
7) Total Years of Experience: ______________

II. Main Interview Themes and sub-Themes:

Q1: Could you please, tell us about Antimicrobial/antibiotic resistance, one of the major public health issues around the world.

Q2: The pharmacy students/future pharmacists at present are ready to play their role in the reduction of AMR/ABR in Pakistan?

Q3: Specific training regarding antibiotic resistance (ABR) and antimicrobial stewardship (ASP) is the need of time in Pakistan?

a.Yes

b. No

3.1: Training, symposiums, conferences, and seminars can help to highlight the ABR issue?

4): Courses related to ABR and ASPs are needed to be a part of the Pharmacy syllabus?

Agree, specify which of the following are being followed.

4.1. The AMR-related subjects at the Pharm-D level are enough? or need to be timely updated, and students must know about the real scenario of AMR.

5. Pharmacy curriculum currently in practice in Pakistan

5.1. The pharmacy syllabus provides adequate education regarding antibiotic use and resistance.

5.2. Do you think the current Pharm-D syllabus is old and to be revised immediately?

5.3. Does the current Pharm-D syllabus cover all the aspects of Antimicrobial resistance?

5.4. The current syllabus doesn't include enough subjects that should cover the AMR.

6. Education and training updated information on AMR

6.1. The Pharmacy institutes provide adequate surveillance and up to date knowledge for drug-resistant organisms?

6.2. Academia and Hospital linkage is the need of time, during Pharm-D internship more focus should be on AMR/ABR.

7. Antibiotic Stewardship Program (ASP)

7.1. How faculty members/teachers of Pharmacy institutions can play role in the ASP implementation?

7.2. Academia and hospitals linkage will reduce the gap in Pakistani?

7.3. Can you please tell us what are the barriers in the ASP implementation in Pakistani hospitals?

7.4. What would be the impact of ASP on the AMR?

7.5. Recommendations for the implementation of stewardship activities in the community?

Any other recommendation________________________________

THANKS FOR PARTICIPATION
